# Differential Association of Viral Dynamics With Disease Severity Depending on Patients’ Age Group in COVID-19

**DOI:** 10.3389/fmicb.2021.712260

**Published:** 2021-07-23

**Authors:** Yuri Kim, Shinhyea Cheon, Hyeongseok Jeong, Uni Park, Na-Young Ha, Jooyeon Lee, Kyung Mok Sohn, Yeon-Sook Kim, Nam-Hyuk Cho

**Affiliations:** ^1^Department of Microbiology and Immunology, College of Medicine, Seoul National University, Seoul, South Korea; ^2^Medical Research Center, Institute of Endemic Diseases, Seoul National University, Seoul, South Korea; ^3^Department of Internal Medicine, School of Medicine, Chungnam National University, Daejeon, South Korea; ^4^Department of Biomedical Sciences, College of Medicine, Seoul National University, Seoul, South Korea; ^5^Seoul National University Bundang Hospital, Seongnam, South Korea

**Keywords:** SARS-CoV-2, COVID-19, viral load, severity, inflammation

## Abstract

Despite a clear association of patient’s age with COVID-19 severity, there has been conflicting data on the association of viral load with disease severity. Here, we investigated the association of viral load dynamics with patient’s age and severity of COVID-19 using a set of respiratory specimens longitudinally collected (mean: 4.8 times/patient) from 64 patients with broad distribution of clinical severity and age during acute phase. Higher viral burden was positively associated with inflammatory responses, as assessed by IL-6, C-reactive protein, and lactate dehydrogenase levels in patients’ plasma collected on the same day, primarily in the younger cohort (≤59 years old) and in mild cases of all ages, whereas these were barely detectable in elderly patients (≥60 years old) with critical disease. In addition, viral load dynamics in elderly patients were not significantly different between mild and critical cases, even though more enhanced inflammation was consistently observed in the elderly group when compared to the younger group during the acute phase of infection. The positive correlation of viral load with disease severity in younger patients may explain the increased therapeutic responsiveness to current antiviral drugs and neutralizing antibody therapies in younger patients compared to elderly patients. More careful intervention against aging-associated inflammation might be required to mitigate severe disease progression and reduce fatality in COVID-19 patients more than 60 years old.

## Introduction

Coronavirus disease 2019 (COVID-19), caused by infection with a new emerging severe acute respiratory syndrome coronavirus-2 (SARS-CoV-2) (Coronaviridae Study Group of the International Committee on Taxonomy of Viruses., 2020), has been rapidly spreading worldwide and has been a global threat to public health since December 2019 (Zhou et al., 2020). Disease mortality, approximately 2.0%, is primarily caused by severe pneumonia and acute respiratory distress syndrome (ARDS) (Yang et al., 2020), whereas around 20% (14–39%) of confirmed cases are asymptomatic (Buitrago-Garcia et al., 2020; Li C. et al., 2021) and most symptomatic people with COVID-19 develop only mild (−40%) or moderate (−40%) disease (Kim et al., 2020; Zhou et al., 2020). Even though the mechanisms underlying this varying degree of pneumonia severity observed in COVID-19 patients remain elusive (Tay et al., 2020), patient’s age is a clear risk factor associated with COVID-19 severity and mortality (Zhou et al., 2020; O’Driscoll et al., 2021). Age-specific fatality rates estimated by an ensemble model range from 0.001% in those aged 5–9 years (range, 0–0.002% across individual national-level seroprevalence surveys) to 8.29% in those over 80 years (range, 2.49–15.55% across individual national-level seroprevalence surveys) (O’Driscoll et al., 2021). COVID-19 fatality in South Korea also gradually increases with patient’s age (0.06% in 30’s, 0.09% in 40’s, 0.29% in 50’s, 1.31% in 60’s, 6.19% in 70’s, and 19.88% in over 80 years)^1^. In addition, it has been reported that viral load in respiratory tracts may also be associated with COVID-19 severity and mortality (Fajnzylber et al., 2020; Magleby et al., 2020; Pujadas et al., 2020; Westblade et al., 2020). However, several other studies have reported no significant difference in viral loads across sex, age, and/or disease severity (He et al., 2020; Madera et al., 2021; Mahallawi et al., 2021). Given that viral dynamics and *in vivo* variation among subgroups may play a significant role in the epidemiological and clinical characteristics of COVID-19, assessing the association of viral load kinetics with disease severity might inform proper therapeutic strategies for mitigating COVID-19 severity and/or mortality, as well as identifying preventative measures for public health. Here, we investigate the association of viral load dynamics with patient’s age and COVID-19 severity using a set of respiratory specimens longitudinally collected from 64 patients with a broad range of clinical severity and age distribution.

## Materials and Methods

### Ethics Statement

The current research was approved by the institutional review boards of Chungnam National University Hospital (IRB no.: CNUH2017–12–004), and Seoul National University Hospital (IRB no.: C-1509-103-705). This study was performed in accordance with the ethical standards laid down in the 1964 Declaration of Helsinki and all subsequent revisions. This study was conducted with informed consent from patients or their legal guardians.

### Patient Groups

General information on the clinical courses and baseline characteristics of the COVID-19 patients included in this study are summarized in Supplementary Table 1 and Table 1. The patients were divided into two groups based on disease severity. The mild group includes 42 patients who had mild respiratory symptoms but no detectable pneumonia, or mild to moderate pneumonia as determined by chest imaging and clinical symptoms. The critical group includes 22 patients who suffered from severe pneumonia and ARDS requiring high flow oxygen supply and/or mechanical ventilation. Among the critical group patients, six patients succumbed to death due to fatal ARDS. Concentration of IL-6, CRP, and LDH in plasma specimens were measured via clinical diagnosis service from Seoul Clinical Laboratory (Seoul, South Korea).

**TABLE 1:** Summary of COVID-19 patients enrolled in this study.

Age group (*n, %*)	Sex (*n, %*)	Severity (*n, %*)
∼29 (9, 14.1)	M (4) F (5)	Mild (9, 14.4) Critical (0, 0.0)
30 ∼ 39 (6, 9.4)	M (5) F (1)	Mild (5, 7.8) Critical (1, 1.6)
40 ∼ 49 (7, 10.9)	M (3)F (4)	Mild (6, 9.4) Critical (1, 1.6)
50 ∼ 59 (9, 14.1)	M (4) F (5)	Mild (7, 10.9) Critical (2, 3.1)
60 ∼ 69 (17, 26.6)	M (10) F (7)	Mild (9, 14.4) Critical (8, 12.5)
70 ∼ 79 (8, 12.5)	M (1)F (7)	Mild (1, 1.6) Critical (7, 10.9)
80 ∼ (8, 12.5)	M (3)F (5)	Mild (5, 7.8) Critical (3, 4.7)
Younger [∼ 59] (31, 48.4)	M (16, 25.0) F (15, 23.4)	Mild (27, 42.2) Critical (4, 6.3)
Older [60 ∼] (33, 51.6)	M (14, 21.9) F (19, 29.7)	Mild (15, 23.4) Critical (18, 28.1)
Total (64, 100.0)	M (30, 46.9) F (34, 53.1)	Mild (42, 65.6) Critical (22, 34.4)

### Quantitation of Viral Loads

Real-time reverse transcription-polymerase chain reaction (RT-PCR) assay for detection of SARS-CoV-2 was performed according to the manufacturer’s instructions (KogeneBiotech, Seoul, South Korea). Total RNAs were obtained from nasopharyngeal swab samples (upper respiratory tract) and sputa (lower respiratory tract). Primer sets targeting E and RdRP genes of SARS-CoV-2 were used with a cut-off cycle threshold (Ct) value of higher than 38 cycles. Viral copies were calculated based on the degree of association between Ct values and Log_10_(viral concentration) as described in the product information and a previous report (Hur et al., 2020). Negative samples were presented as −1 of Log_10_(viral concentration) in graphs.

### Statistical Analysis

Data was analyzed using the Graph Pad Prism 5.01 software (GraphPad Software, La Jolla, CA, United States) and Microsoft Excel (Microsoft Office Professional Plus 2016). Statistical analyses were performed using a two-tailed Mann–Whitney *U* test, or Kruskal–Wallis test with Dunn’s multiple comparisons among different groups. Spearman’s rank test was used to analyze the correlation between variables. A *p*-value of <0.05 was considered statistically significant.

## Results

### Differential Association of Viral Load With Patient’s Age and COVID-19 Severity

Baseline characteristics of the confirmed 64 COVID-19 patients included in this study are summarized in Table 1 and Supplementary Table 1. The mild group includes 42 (65.6%) patients who had mild respiratory symptoms but no detectable pneumonia, or showed mild to moderate pneumonia as determined by chest imaging and clinical symptoms. The critical group includes 22 (34.4%) patients who suffered from severe pneumonia and ARDS requiring high flow oxygen supply and/or mechanical ventilation. Among the critical patients, sixteen patients survived and were discharged, while six patients succumbed to death due to fatal ARDS.

**FIGURE 1:**
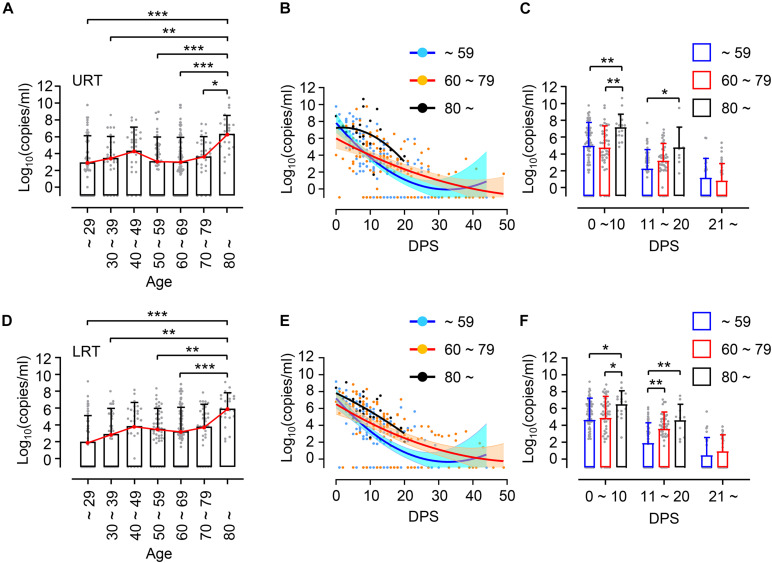
Distribution and kinetic changes of respiratory viral loads in different age groups. **(A)** Distribution of viral loads in nasopharyngeal swabs representing upper respiratory tract (URT) specimens from the indicated age groups (∼ 29: *n* = 54, 30 ∼ 39: *n* = 31, 40 ∼ 49: *n* = 28, 50 ∼ 59: *n* = 44, 60 ∼ 69: *n* = 92, 70 ∼ 79: *n* = 31, 80 ∼: *n* = 24) were evaluated by quantitative RT-PCR targeting the E gene of SARS-CoV-2. **(B)** Kinetic changes of viral loads in URT samples from the indicated age groups are presented. The blue, red, and black lines show the trend in viral loads of the indicated age groups, using curve fit with non-linear regression with 95% confidence intervals (shaded color) from the regression line. Blue (∼ 59): *n* = 157, Red (60 ∼ 79): *n* = 123, Black (80 ∼): *n* = 24. **(C)** Distribution of viral loads in URT samples from the indicated age groups are presented (mean + SD) during the indicated period. **(D)** Distribution of viral loads in sputa representing lower respiratory tract (LRT) specimens from the indicated age groups (∼ 29: *n* = 54, 30 ∼ 39: *n* = 31, 40 ∼ 49: *n* = 28, 50 ∼ 59: *n* = 44, 60 ∼ 69: *n* = 118, 70 ∼ 79: *n* = 34, 80 ∼: *n* = 24). **(E)** Kinetic changes of viral loads in LRT samples from the indicated age groups as shown in **(B)**. Blue (∼ 59): *n* = 157, Red (60 ∼ 79): *n* = 152, Black (80 ∼): *n* = 24. **(F)** Distribution of viral loads in LRT samples from the indicated age groups are presented (mean + SD) at the indicated period. DPS, days post symptom onset. Statistical significance in difference among the groups were assessed by Kruskal–Wallis test for comparisons of values among different groups. *p < 0.05, **p < 0.01, ***p < 0.001.

First, we investigated the potential association of viral loads in respiratory specimens, nasopharyngeal swabs as upper respiratory tract (URT) samples and sputa as lower respiratory tract (LRT) samples, with patient’s age. Enrolled patients were grouped by 10-year intervals: ages ∼ 29 (*n* = 9, 14.1%), 30 ∼ 39 (*n* = 6, 9.4%), 40 ∼ 49 (*n* = 7, 10.9%), 50 ∼ 59 (*n* = 9, 14.1%), 60 ∼ 69 (*n* = 17, 26.6%), 70 ∼ 79 (*n* = 8, 12.5%), and over 80 (*n* = 8, 12.5%) (Table 1). Due to the small sample sizes using 10 years brackets, we also categorized the patients into a younger group (less than 60 years, *n* = 31, 48.4%) and elderly group (60 years and over, *n* = 33, 51.6%). When we compared virus shedding patterns in individual volunteers with regressed viral load lines to examine biased effect of data pooling (Hooker and Ganusov, 2021), there was a reasonable correspondence between regression lines of overall viral loads and shedding for individual volunteers (Supplementary Figure 1). The distribution of assessed viral loads in a total of 304 URT specimens and 306 LRT samples longitudinally collected from the patients are presented by age group in Figure 1. Although there is a variation of mean viral loads among 10-year interval age groups (mean log_10_ viral load in URT: 2.86 ∼ 6.35 and in LRT: 1.98 ∼ 6.92), we only observed a significant difference in the group over 80 years old, which showed the highest mean viral loads in both URT (mean log_10_ viral load = 6.35) and LRT (mean log_10_ viral load = 6.92) specimens. Viral RNA in respiratory specimens during the first 20 days (D0 ∼ D20) after symptom onset were generally higher in older patients, particularly those over 80 years old, when compared to the younger group under 60 years old (Figures 1B,C,E,F). In addition, significantly higher viral loads were also observed in LRT from the 60 ∼ 79 years old group during the second 10 days (D11 ∼ D20) after symptom onset, when compared to those from the younger age group (Figures 1E,F).

**FIGURE 2:**
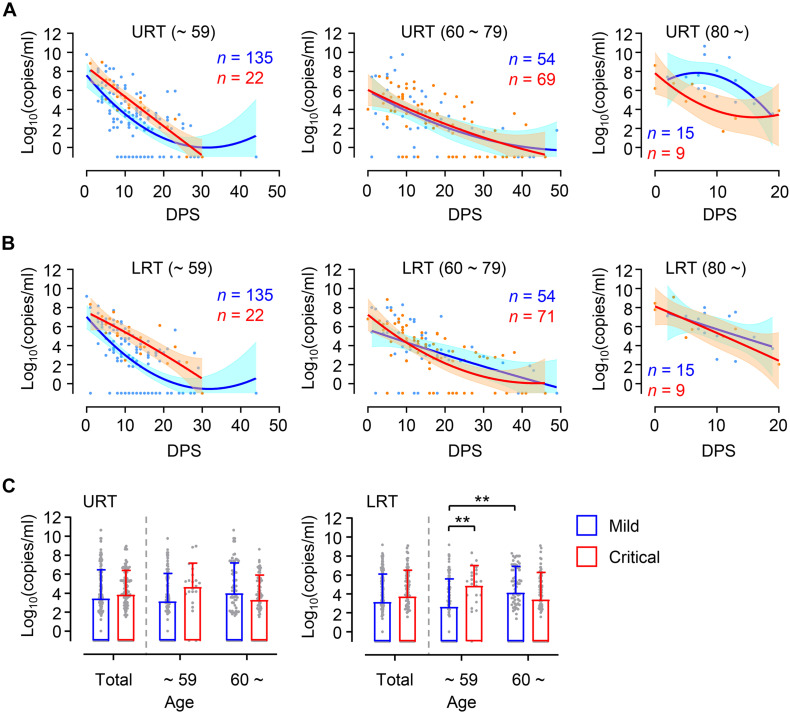
Differential kinetics of respiratory viral load in young and old age groups. **(A,B)** Kinetic changes of viral loads in URT **(A)** or LRT **(B)** samples from the indicated age groups are presented. The blue and red lines show the trend in viral loads of mild and critical groups, respectively, using curve fit with non-linear regression with 95% confidence intervals (shaded color) from the regression line. The number of specimens are presented in the corresponding color. DPS, days post symptom onset. **(C)** Distribution of viral loads in URT (left) and LRT (right) samples from the indicated age groups are presented (mean + SD) at the indicated period. **p < 0.01 (two-tailed Mann–Whitney test for pairwise comparison between mild and critical groups in total or Kruskal–Wallis test for comparisons of values among different age groups).

Higher viral loads in elderly patients might be due to increased prevalence of severe COVID-19 disease compared to younger patients (Table 1). To confirm this, we assessed viral loads in younger and elderly patient groups after dividing them based on disease severity (Figure 2). During the acute phase of infection, relatively higher viral loads were consistently observed in both URT and LRT specimens from critical patients than those from mild patients in the younger group. Viral loads in critical cases of younger age group were significantly higher than those of mild group in LRT. In contrast, kinetic responses and overall viral loads in elderly patients were not significantly different between mild and critical cases. Interestingly, we observed more elevated and sustained viral release in URT specimens from mild patients over 80 years old when compared to those from critical cases, but kinetic responses of viral loads in LRT samples were similar regardless of disease severity. Even though significantly higher viral loads were observed in LRT specimens from the elderly group with mild disease when compared to those in mild COVID-19 patients of the younger group, viral loads observed in the all the specimens from URT and LRT were not statistically different between mild and severe cases in the collective patient cohort (Figure 2C, Total). These results indicate that viral responses in COVID-19 patients might be differentially regulated according to patient’s age, wherein disease severity is associated with higher viral loads only in younger patients and not in elderly patients. When we analyzed the kinetic responses of viral loads for all patients according to disease severity, there was a non-significantly higher viral load in critical cases, especially in LRT specimens during the acute phase (D0 ∼ D20 after symptom onset) (Supplementary Figures 2A,B). This moderate difference in viral loads between mild and critical cases is primarily due to elevated viral loads in mild elderly patients (Figure 2). Nevertheless, the levels of inflammatory indicators, including IL-6, C-reactive protein (CRP), and lactate dehydrogenase (LDH) in patients’ plasma, were consistently higher in critical and fatal cases when compared to mild COVID-19 cases throughout the acute phase of infection (Supplementary Figures 2C–E).

### Differential Association of Viral Loads With Inflammatory Indicators Depending on Disease Severity and Patient’s Age

**FIGURE 3:**
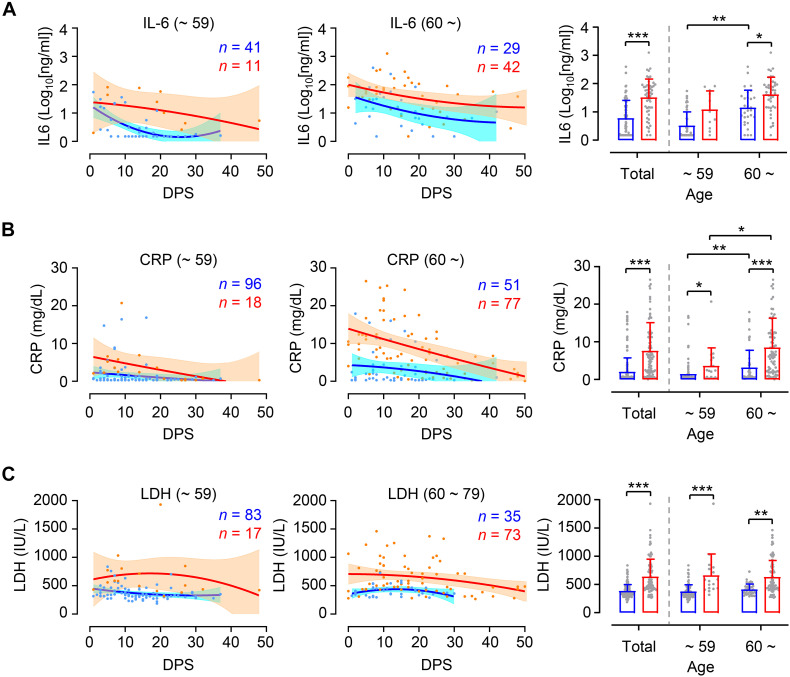
Differential kinetics and levels of inflammatory indicators in plasma from young and old age groups. Kinetic changes of IL-6 **(A)**, CRP **(B)**, and LDH **(C)** levels in plasma samples from the indicated age groups (young: left panels, old: middle panels) are presented. The blue and red lines show the trend in levels of plasma markers of mild and critical groups, respectively, using curve fit with non-linear regression with 95% confidence intervals (shaded color) from the regression line. The number of specimens are presented in the corresponding color. Distribution of plasma markers’ level from the indicated age groups are also presented (mean + SD) in right panels. DPS, days post symptom onset. *p < 0.05, **p < 0.01, ***p < 0.001 (two-tailed Mann–Whitney test for pairwise comparison between mild and critical groups in total or Kruskal–Wallis test for comparisons of values among different age groups).

In order to investigate whether levels of inflammation are different between younger and elderly patients according to disease severity, we assessed kinetic responses of inflammatory indicators, including IL-6, CRP, and LDH in patients’ plasma (Guan et al., 2020; Zhou et al., 2020). As expected, kinetic analysis of these indicators in plasma revealed more elevated and sustained responses in critical cases than in mild group throughout disease course (Figures 3A–C, left and middle panels). Pairwise comparison of mild and critical groups showed relatively increased inflammatory markers in critical cases regardless of age group (Figures 3A–C, right panels). It is also notable that levels of IL-6 and CRP were significantly higher in critical elderly cases (IL-6 mean ± SD: 101.9 ± 200.6 ng/ml, CRP mean ± SD: 8.5 ± 7.8 mg/dl) compared to younger cases (IL-6 mean ± SD: 28.3 ± 31.4 ng/ml, CRP mean ± SD: 3.6 ± 5.8 mg/dl), but also in mild cases (IL-6 mean ± SD in younger and elderly group: 7.3 ± 11.7 and 41.3 ± 82.8 ng/ml, respectively; CRP mean ± SD in younger and elderly group: 1.4 ± 3.0 and 3.2 ± 4.6 mg/dl, respectively). In addition, similar to kinetic responses of viral loads in respiratory secretions, the levels of IL-6 and CRP in plasma peaked during the early phase of symptom onset and gradually declined thereafter regardless of patient’s age and disease severity. However, kinetics of LDH levels in plasma showed rather sustained responses up to D30 after symptom onset, especially in critical COVID-19 patients. Levels of LDH were similar between the age groups in both mild cases (mean ± SD in younger and elderly group: 373.2 ± 118.4 and 408.7 ± 98.7 IU/L, respectively) and critical cases (mean ± SD in younger and elderly group: 660.2 ± 380.3 and 627.8 ± 297.3 IU/L, respectively), although these were significantly different between mild and critical patients.

**FIGURE 4:**
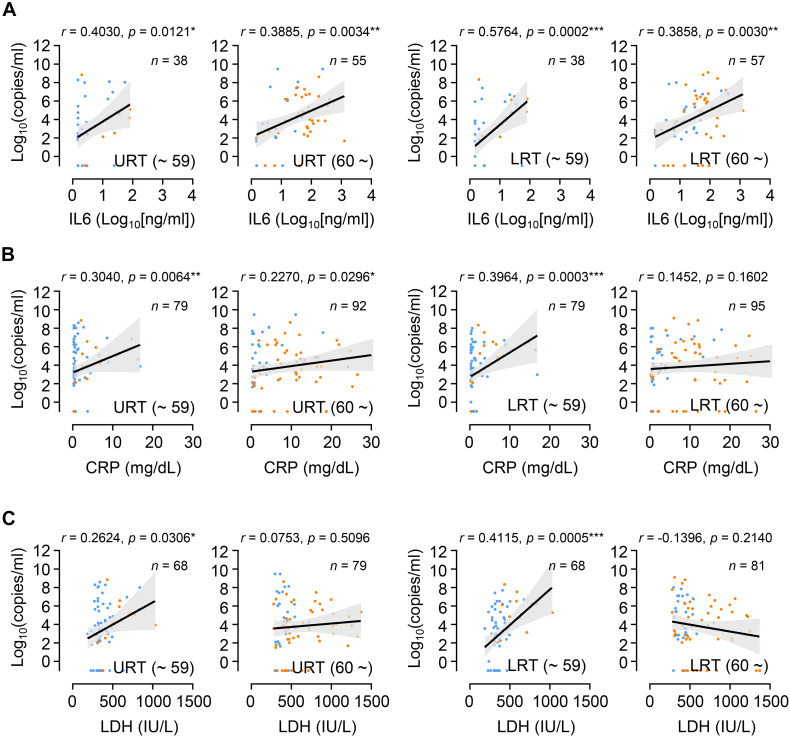
Correlation of respiratory viral load with inflammatory indicators in plasma from young and old age groups. Correlation of IL-6 **(A)**, CRP **(B)**, and LDH **(C)** levels in plasma with respiratory viral loads (URT or LRT) was assessed in paired plasma and respiratory specimens collected on the same day after symptom onset. Linear regression (black line with 95% confidence intervals, shaded color) and Spearman’s rank test were performed to examine statistical significance. Correlation coefficient (*r*) and *p*-values are indicated within the graphs. Total number of paired specimens and age group are presented in each plot. Blue dots: samples from mild cases, orange dots: samples from critical cases.

Since we observed similar kinetics of viral responses in respiratory tracts and inflammatory responses represented by the levels of IL-6 and CRP in plasma, regardless of patient’s age and disease severity, we assessed the potential correlation of viral loads in respiratory specimens and the levels of inflammatory indicators in plasma collected on the same day after symptom onset. Overall viral loads observed in URT or LRT specimens correlated significantly with the levels of IL-6, CRP, and LDH in paired plasma collected on the same day after symptom onset (Supplementary Figure 3). However, after categorizing the data based on age group, the positive correlation between viral loads in respiratory tract specimens and inflammatory indicators in plasma was generally more significant in younger patients than in elderly patients (Figure 4). Therefore, viral loads measured in respiratory secretions during the symptomatic period are significantly associated with systemic inflammation in younger patients, but seems to be weaker or not significant in the elderly patients.

The differential dynamics of viral loads observed in our COVID-19 cohort could also be associated with therapeutic treatment with various anti-viral drugs and corticosteroids (Supplementary Table 1; Tarighi et al., 2021). Therefore, we examined respiratory viral dynamics after categorizing patients by anti-viral (Supplementary Figure 4) or corticosteroid treatment (Supplementary Figure 5). Although there was rather delayed viral clearance in patients administered corticosteroids, it was not significantly different when compared with non-treated group, as previously reported (Li S. et al., 2021). Younger patients treated with corticosteroids showed relatively higher viral loads and delayed clearance in LRT, but this was barely observed in URT specimens (Supplementary Figure 5). The difference in viral dynamics between treated and non-treated patients was not significant in the older age group. With regards to anti-viral drugs, we failed to detect a significant difference between treated and non-treated groups regardless of patient’s age (Supplementary Figure 4). URT specimens from the younger group treated with anti-viral drugs showed more rapid viral clearance, but without statistical significance. Despite confounding variation in types of anti-viral drugs, co-administration of corticosteroids, time of drug administration, and disease severity at the time of treatment (Tarighi et al., 2021), differences in viral dynamics and burden in respiratory tracts due to drug treatment were generally more obvious in younger age group than elderly patients.

## Discussion

Despite a clear association of patient’s age with COVID-19 severity, there has been conflicting data on the association of viral load with disease severity. Several studies have reported higher SARS-CoV-2 viral loads in respiratory tracts and/or plasma with disease severity and old age (Fajnzylber et al., 2020; Magleby et al., 2020; Pujadas et al., 2020; Westblade et al., 2020; Maltezou et al., 2021), but others have reported no obvious difference between viral loads and disease severity (He et al., 2020; Jacot et al., 2020). These contradictory results might be due to variations in sampling time points after symptom onset, sample collection from different respiratory sites, distribution of patient’s age, gender, and comorbidity, as well as treatment with different anti-viral and anti-inflammatory medications. To reduce the effect of these confounding factors, we longitudinally collected respiratory samples (1 ∼ 10 times after symptom onset, average of 4.8 times/patient) and used the same types of respiratory specimens (nasopharyngeal swabs and sputa). We also categorized the patients by disease severity (mild symptom vs. critical ARDS requiring high flow oxygen supply and/or mechanical ventilation) and age (younger age group less than 60 years old vs. elderly group 60 years and over) because mortality sharply increases to over 1% in elderly patients 60 years old and over (O’Driscoll et al., 2021). Although we failed to observe significant differences in viral loads among 10-year interval age groups, except those over 80 who showed the highest mean viral loads with delayed viral clearance (Figure 1), there was an obvious difference in viral load dynamics between younger (≤59) and elderly (≥60) groups in association with disease severity (Figure 2). In the younger age group, viral loads in LRT were significantly higher in critical cases than those of mild patients during the acute phase of infection. However, this difference was barely observed in the elderly group. Moreover, we noted higher viral loads with delayed clearance in URT specimens from patients over 80 years old with mild disease than those from critical disease although the sample size is rather small (mild: *n* = 15 from five patients, critical: *n* = 9 from three patients). In addition, viral loads in LRT from mild elderly cases were significantly higher than those from younger age group (Figure 2C). These results clearly indicate that viral loads in respiratory tracts of COVID-19 patients are positively associated with disease severity in younger age group, but barely in elderly patients. Consistently, respiratory viral loads generally correlated with inflammatory markers in younger patients, but less correlated with those in older patients (Figure 4). When we assessed the correlation of viral loads with disease severity, there was a significant positive correlation only in mild cases, but not in severe and fatal groups (Supplementary Figure 6). Therefore, respiratory viral burden seems to be positively associated with inflammatory responses mainly in younger aged group and mild cases, but barely in elderly patients with severe disease.

Positive correlation of viral load with disease severity in younger patient group may explain why therapeutic responsiveness was significantly better in younger age group than elderly patients who received an anti-viral drug, such as remdesivir (Beigel et al., 2020), or convalescent immune plasma (Joyner et al., 2021), as determined by viral load and disease severity. More enhanced inflammatory responses, as evidenced by elevated IL-6 and CRP levels in plasma, were consistently observed in elderly patients than younger group even soon after symptom onset (Figure 3) and viral burden is less likely to be associated with disease severity in the elderly. These suggest that intrinsic nature of overt inflammation, rather than direct viral insult, in elderly patients might be responsible for severe pulmonary disease. Therefore, more careful intervention against aging-associated inflammation might be required to mitigate critical disease progression and reduce fatality in COVID-19 patients more than 60 years old. Since immunosenescence and inflammaging have been considered key features contributing to increased inflammatory phenotypes causing immune dysfunction, improved understanding of the pathophysiology of aging and the pulmonary inflammation associated with COVID-19 will not only help understand the underlying mechanisms of severe COVID-19 but also guide targeted management strategies for this emerging viral disease (Akbar and Gilroy, 2020; Bajaj et al., 2020).

## Data Availability Statement

The original contributions presented in the study are included in the article/Supplementary Material, further inquiries can be directed to the corresponding author/s.

## Ethics Statement

The studies involving human participants were reviewed and approved by the Chungnam National University Hospital (IRB no.: CNUH2017–12–004), and Seoul National University Hospital (IRB no.: C-1509-103-705). The patients/participants provided their written informed consent to participate in this study.

## Author Contributions

Y-SK and N-HC conceptualized the study. YK, SC, Y-SK, and N-HC designed the methodology and wrote the manuscript. YK, SC, HJ, UP, N-YH, JL, KS, Y-SK, and N-HC conducted the investigation. SC, JL, KS, and Y-SK provided resources. N-HC provided funding. All authors contributed to the article and approved the submitted version.

## Conflict of Interest

The authors declare that the research was conducted in the absence of any commercial or financial relationships that could be construed as a potential conflict of interest.

## Publisher’s Note

All claims expressed in this article are solely those of the authors and do not necessarily represent those of their affiliated organizations, or those of the publisher, the editors and the reviewers. Any product that may be evaluated in this article, or claim that may be made by its manufacturer, is not guaranteed or endorsed by the publisher.

## Footnotes

1
https://covid19.kdca.go.kr/

## Supplementary Material

Supplementary Figure 1 **(A,B)** Kinetic changes of viral loads in URT **(A)** and LRT **(B)** samples from all the patients are presented. Viral loads in individual volunteers is shown by gray lines and the regression lines show the trend, using curve fit with linear regression with 95% confidence intervals (dotted lines). The number of specimens and *r*^2^ value from regression analysis are presented. DPS, days post symptom onset.

Supplementary Figure 2 **(A,B)** Kinetic changes of viral loads in URT **(A)** and LRT **(B)** samples from mild (blue), critical (red), and fatal (black) groups are presented. The critical group data includes fatal cases. The regression lines in left panels show the trend, using curve fit with non-linear regression with 95% confidence intervals (shaded color). The number of specimens are presented in the corresponding color. DPS, days post symptom onset. **(C–E)** Kinetic changes of IL-6 **(C)**, CRP **(D)**, and LDH **(E)** in patient’s plasma from mild (blue), critical (red), and fatal (black) groups are presented. The critical group data includes fatal cases. The regression lines in left panels show the trend, using curve fit with non-linear regression with 95% confidence intervals (shaded color). The number of specimens are presented in the corresponding color. DPS, days post symptom onset. **p* < 0.05, ***p* < 0.01, ****p* < 0.001 (two-tailed Mann–Whitney test).

Supplementary Figure 3 Correlation of respiratory viral load with inflammatory indicators in plasma from COVID-19 patients. Correlation of IL-6 **(A)**, CRP **(B)**, and LDH **(C)** levels in plasma with respiratory viral loads (URT or LRT) was assessed in paired plasma and respiratory specimens collected on the same day after symptom onset. Linear regression (black line with 95% confidence intervals, shaded color) and Spearman’s rank test were performed to examine statistical significance. Correlation coefficient (*r*) and *p*-values are indicated within the graphs. Total number of paired specimens and age group are presented in each plot.

Supplementary Figure 4 Kinetic response of respiratory viral load in COVID-19 patients treated with anti-viral drugs. **(A,B)** Kinetic changes of viral loads in URT **(A)** or LRT **(B)** samples from the indicated age groups are presented. The blue and red lines show the trend in viral loads in non-treated and treated patients with various anti-viral drugs (see Supplementary Table 1), respectively, using curve fit with non-linear regression with 95% confidence intervals (shaded color) from the regression line. We used all the available data sets regardless of treatment period. DPS, days post symptom onset. **(C)** Distribution of viral loads in URT (left) and LRT (right) samples from the indicated age groups treated or non-treated conditions are presented (mean + SD).

Supplementary Figure 5 Kinetic response of respiratory viral load in COVID-19 patients treated with corticosteroid drugs. **(A,B)** Kinetic changes of viral loads in URT **(A)** or LRT **(B)** samples from the indicated age groups are presented. The blue and red lines show the trend in viral loads in non-treated and treated patients with various corticosteroid drugs (see Supplementary Table 1), respectively, using curve fit with non-linear regression with 95% confidence intervals (shaded color) from the regression line. We used all the available data sets regardless of treatment period. DPS, days post symptom onset. **(C)** Distribution of viral loads in URT (left) and LRT (right) samples from the indicated age groups treated or non-treated conditions are presented (mean + SD). Statistical significance was assessed by two-tailed Mann–Whitney test for pairwise comparison or Kruskal–Wallis test for comparisons of values among different age groups. ***p* < 0.01, ****p* < 0.001.

Supplementary Figure 6 Differential correlation of respiratory viral load with inflammatory indicators in plasma according to disease severity. Correlation of IL-6 **(A)**, CRP **(B)**, and LDH **(C)** levels in plasma with respiratory viral loads (URT or LRT) was assessed in paired plasma and respiratory specimens collected from mild (blue), critical (red), and fatal (black) cases on the same day after symptom onset. The critical group data includes fatal cases. Linear regression (colored line with 95% confidence intervals, shaded color) and Spearman’s rank test were performed to examine statistical significance. Correlation coefficient (*r*) and *p*-values are indicated within the graphs (colored correspondingly).

Supplementary Table 1 Baseline characteristics of COVID-19 patients included in this study.
