# Corrigendum: Differential association of viral dynamics with disease severity depending on patients' age group in COVID-19

**DOI:** 10.3389/fmicb.2023.1178685

**Published:** 2023-03-14

**Authors:** Yuri Kim, Shinhyea Cheon, Hyeongseok Jeong, Uni Park, Na-Young Ha, Jooyeon Lee, Kyung Mok Sohn, Yeon-Sook Kim, Nam-Hyuk Cho

**Affiliations:** ^1^Department of Microbiology and Immunology, College of Medicine, Seoul National University, Seoul, South Korea; ^2^Medical Research Center, Institute of Endemic Diseases, Seoul National University, Seoul, South Korea; ^3^Department of Internal Medicine, School of Medicine, Chungnam National University, Daejeon, South Korea; ^4^Department of Biomedical Sciences, College of Medicine, Seoul National University, Seoul, South Korea; ^5^Seoul National University Bundang Hospital, Seongnam, South Korea

**Keywords:** SARS-CoV-2, COVID-19, viral load, severity, inflammation

In the published article, there was a mistake in the **Funding** statement. The grant number for the National Research Foundation of Korea was displayed as “2021M3A9H5020761.” The correct statement is below:

