# Supplementary material for: Differential Association of Viral Dynamics With Disease Severity Depending on Patients’ Age Group in COVID-19
Source: Front Microbiol. 2021 Jul 23;12:712260. doi: 10.3389/fmicb.2021.712260 (PMC8343133; doi:10.3389/fmicb.2021.712260)
Supplement: Supplementary Figure 1 — (A,B) Kinetic changes of viral loads in URT (A) and LRT (B) samples from all the patients are presented. Viral loads in individual volunteers is shown by gray lines and the regression lines show the trend, using curve fit with linear regression with 95% confidence intervals (dotted lines). The number of specimens and r2 value from regression analysis are presented. DPS, days post symptom onset. [file Data_Sheet_1.pdf]

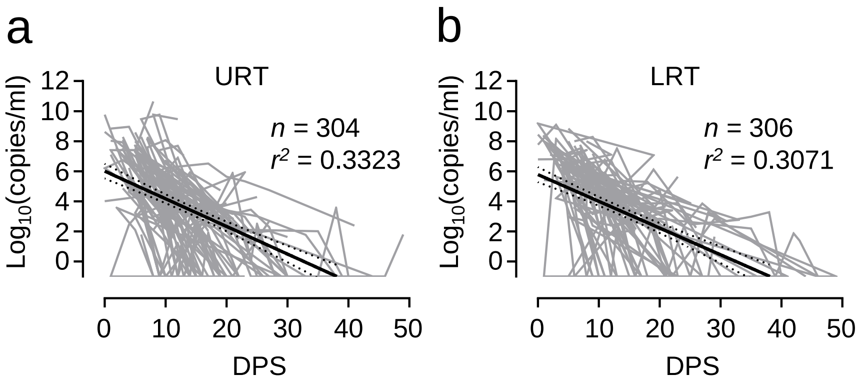

Supplementary Figure S1

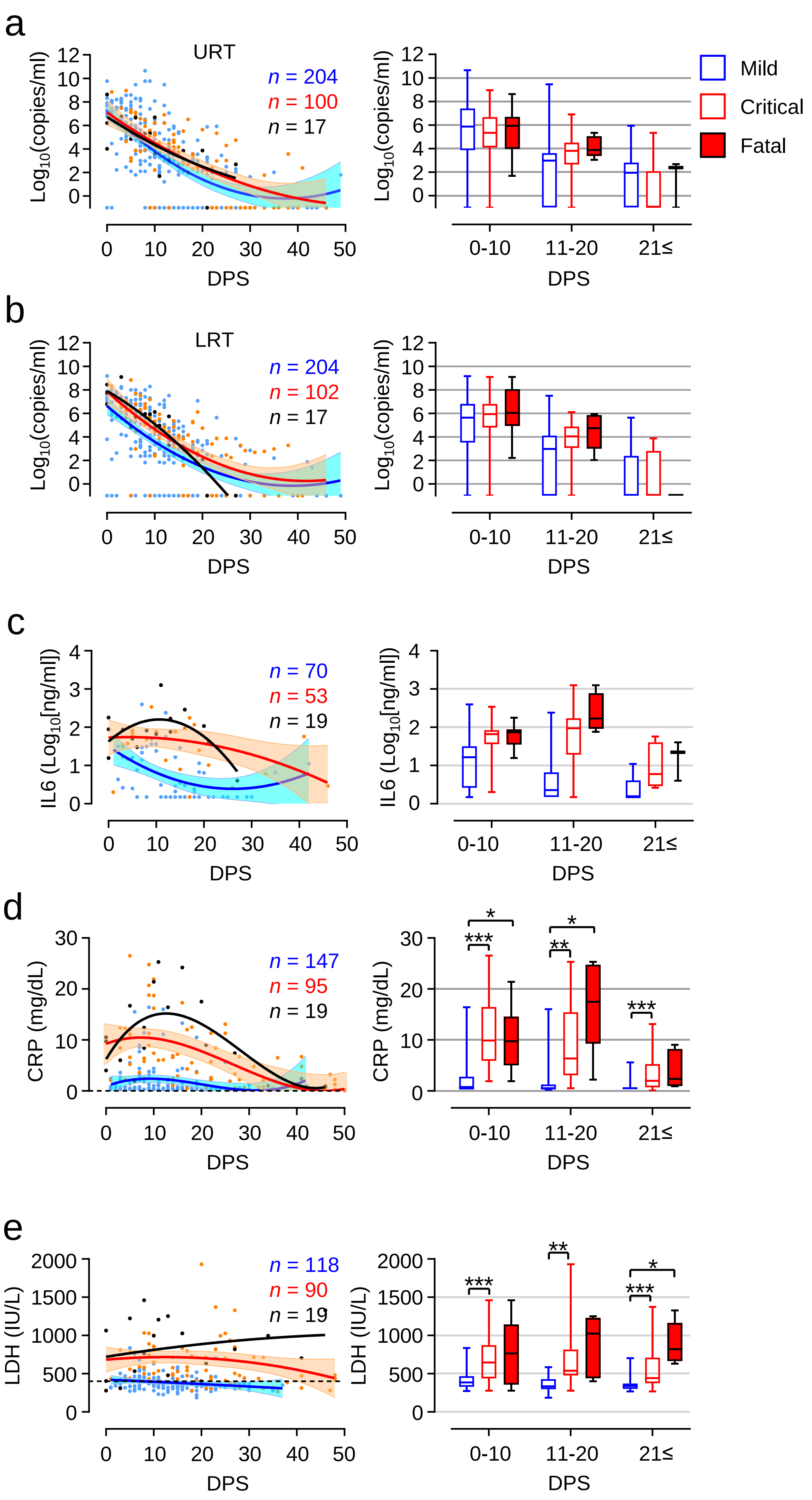

Supplementary Figure S2

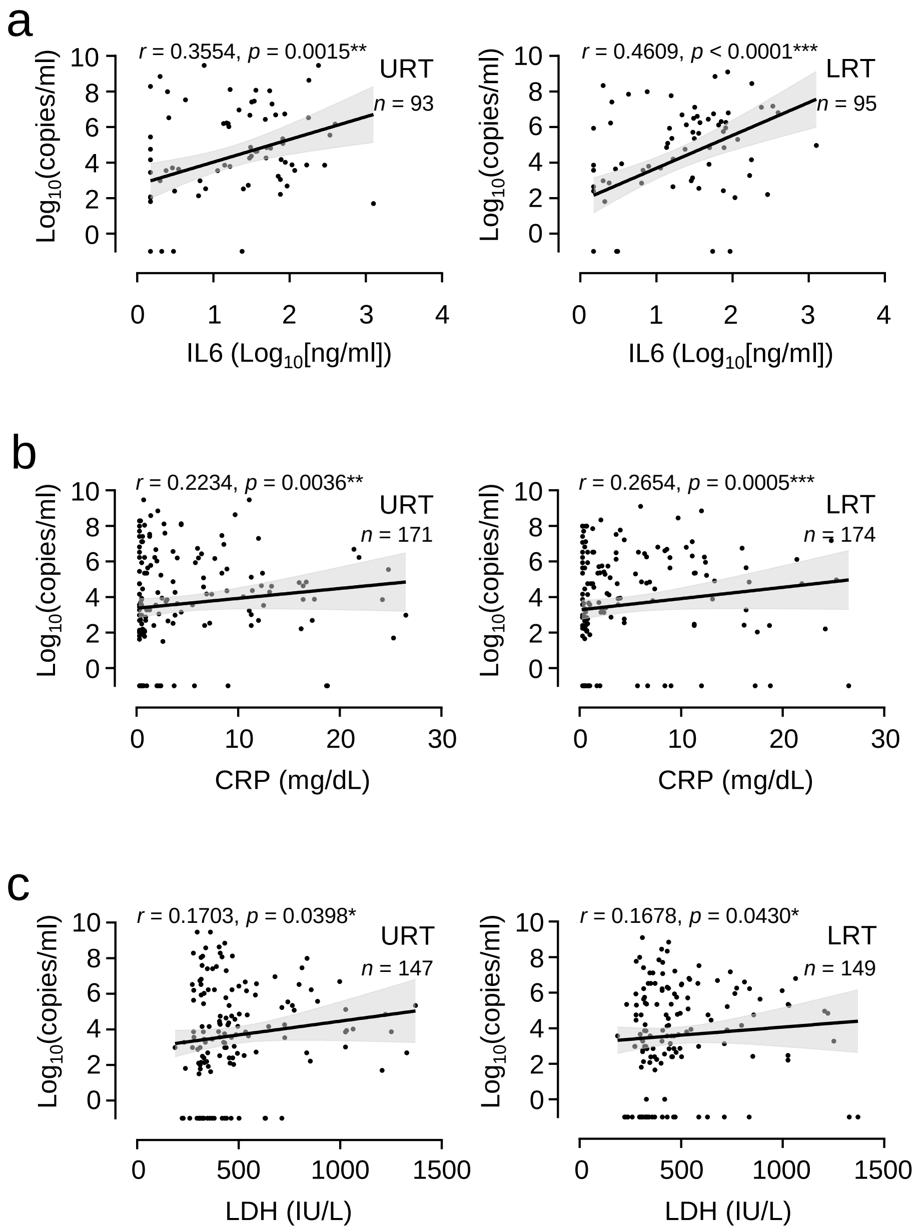

Supplementary Figure S3

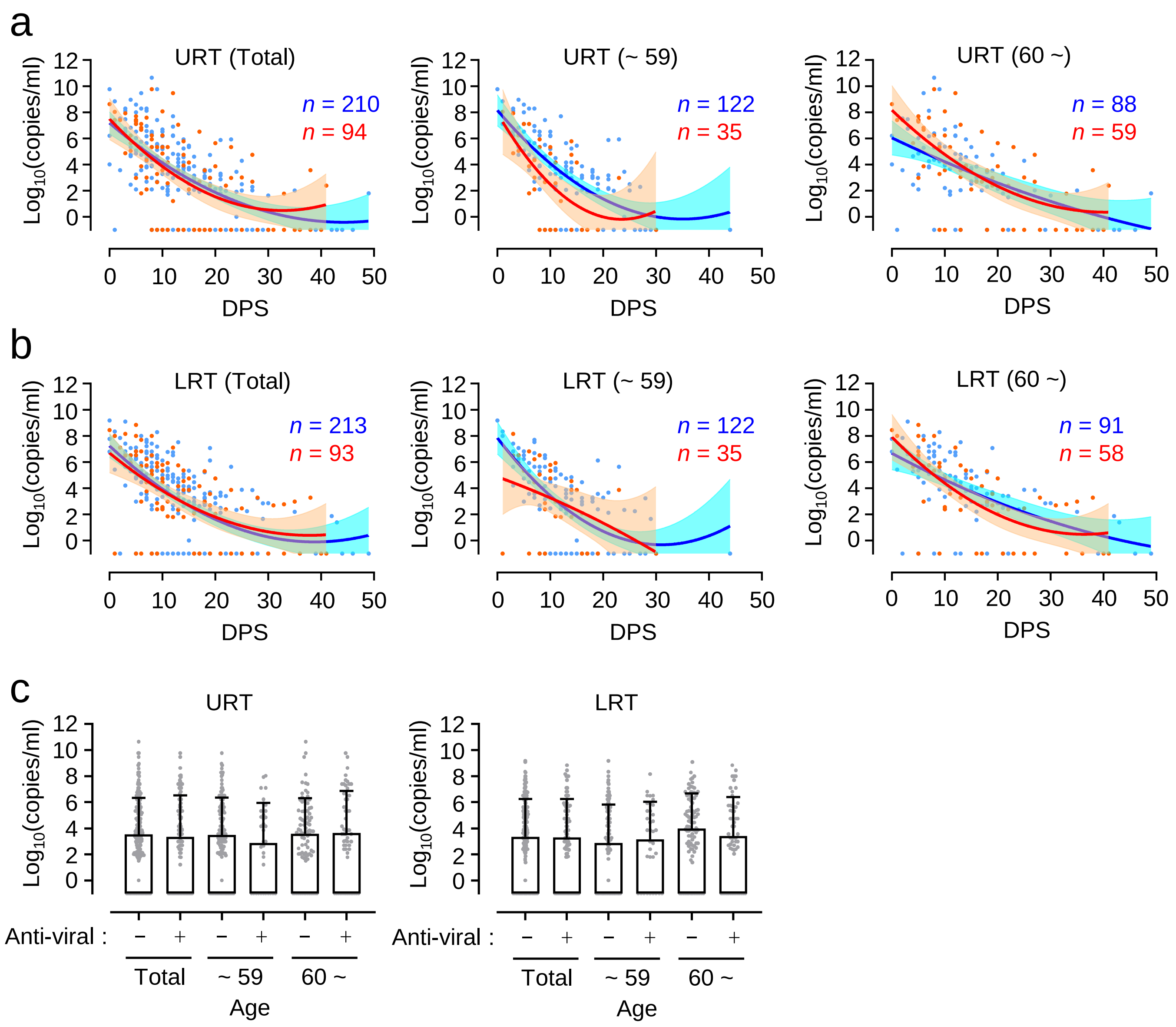

Supplementary Figure S4

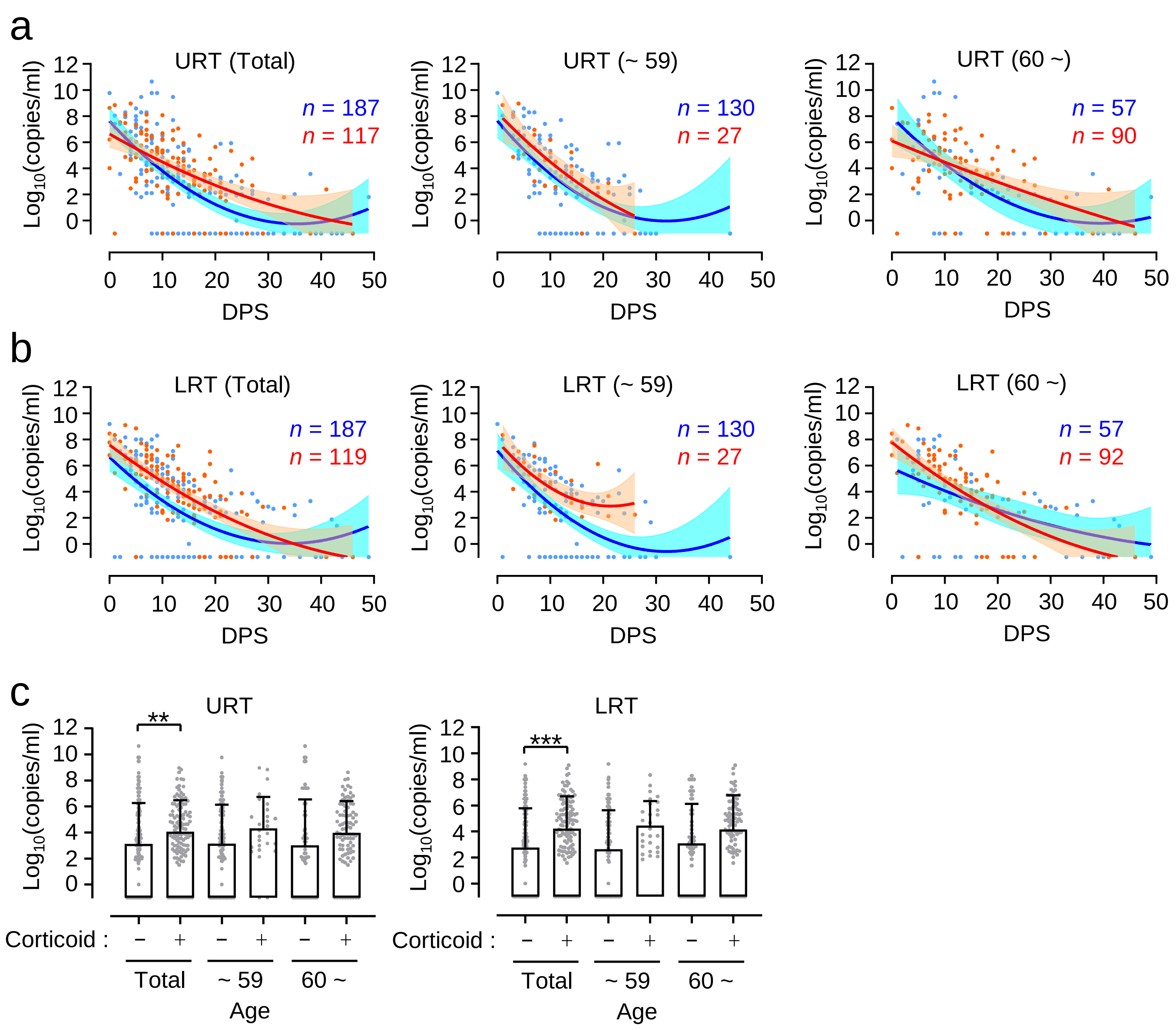

Supplementary Figure S5

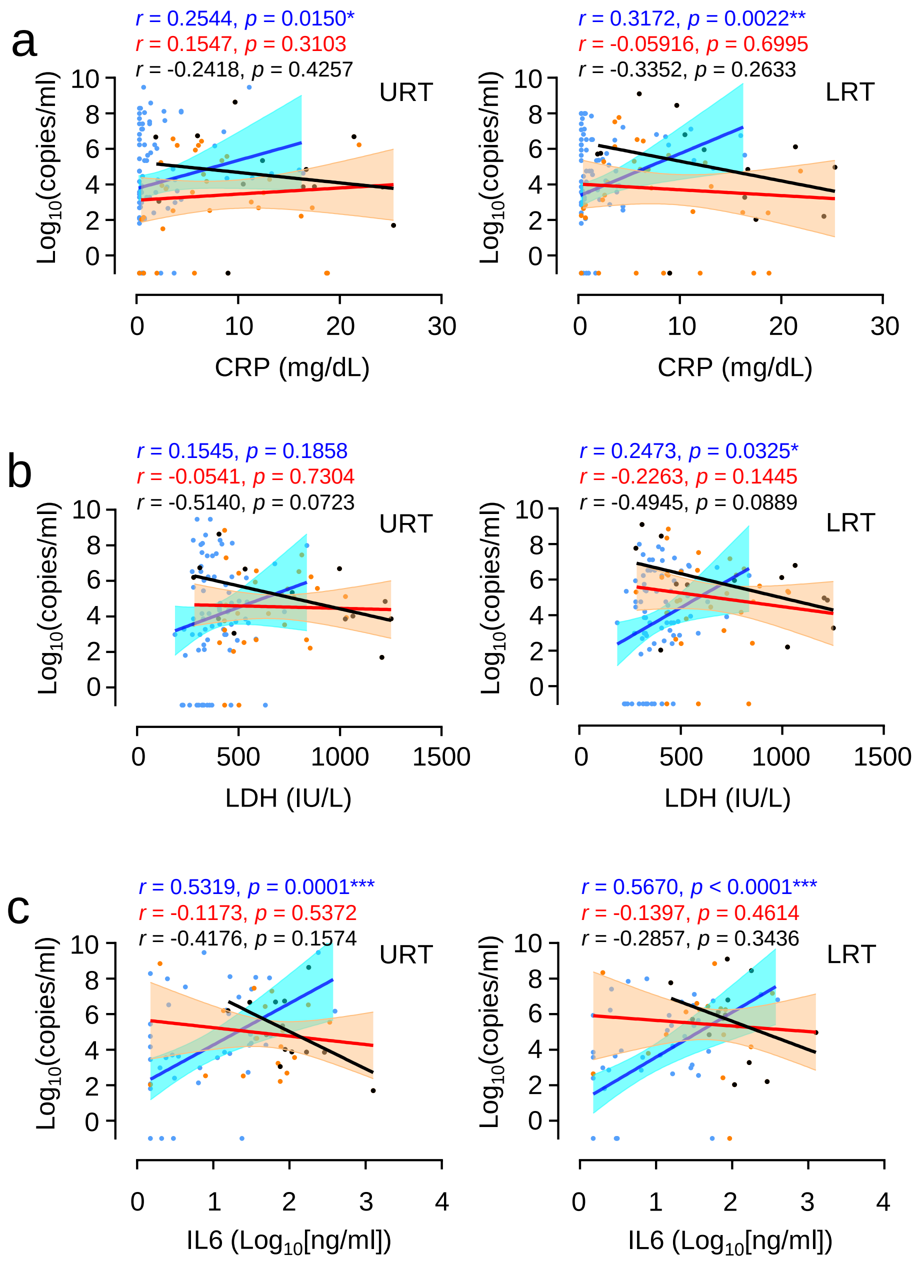

Supplementary Figure S6
